# Associations Between Polymorphisms of Genes Related to Vitamin D Pathway and the Response to Vedolizumab and Ustekinumab in Inflammatory Bowel Disease

**DOI:** 10.3390/jcm13237277

**Published:** 2024-11-29

**Authors:** Jessica Cusato, Davide Giuseppe Ribaldone, Antonio D′Avolio, Valentina Infusino, Miriam Antonucci, Gian Paolo Caviglia, Angelo Armandi, Linda Ceccarelli, Francesco Costa, Andrea Bottari, Pietro Fe, Lorenzo Bertani, Francesca De Vita

**Affiliations:** 1 Department of Medical Sciences, University of Turin, 10126 Turin, Italy; jessica.cusato@unito.it (J.C.); antonio.davolio@unito.it (A.D.); valentina.infusin@edu.unito.it (V.I.); gianpaolo.caviglia@unito.it (G.P.C.); angelo.armandi@unito.it (A.A.); 2 SCDU Infectious Diseases, Amedeo di Savoia Hospital, ASL Città di Torino, 10149 Turin, Italy; miriam.antonucci@aslcittaditorino.it; 3 Azienda Ospedaliera Universitaria Pisana, 56126 Pisa, Italy; ceccarellilinda@gmail.com (L.C.); fcosta@med.unipi.it (F.C.); bottariandrea1@gmail.com (A.B.); pietrofe1996@gmail.com (P.F.); lorenzobertani@gmail.com (L.B.)

**Keywords:** biologics, target therapy, Crohn’s disease, ulcerative colitis, anti-integrin, alpha4beta7, anti-IL12/23, advanced therapy, prediction, tailored therapy

## Abstract

**Background/Objectives**: Vitamin D (VD) has immunoregulatory properties, generating interest in its potential to influence therapeutic outcomes in inflammatory bowel disease (IBD), other than affecting the expression of genes encoding enzymes and transporters involved in drug metabolism and transport. This study investigated VD-related single nucleotide polymorphisms (SNPs) as predictors of clinical responses in patients with Crohn’s disease (CD) and ulcerative colitis (UC) treated with vedolizumab (VDZ) or ustekinumab (UST) after 3 (T3) and 12 months (T12), as well as the achievement of fecal calprotectin (FC) levels < 250 mg/kg, a marker of mucosal healing. **Methods**: In this prospective study, 103 patients (67 CD, 36 UC) were enrolled, 40 receiving VDZ and 63 receiving UST. SNPs in the genes *CYP24A1*, GC, *CYP27B1*, and VD receptor (*VDR*) were analyzed via polymerase chain reaction (PCR) and associated with clinical and laboratory outcomes. **Results**: UST therapy demonstrated a higher clinical response rate at T12 compared to VDZ (*p* = 0.03). A correlation was found between response at T3 and T12 (*p* = 0.0002). GC 1296 AC polymorphism negatively predicted response at T12, with 63.6% of non-responders carrying this genotype. *CYP24A1* 8620 AG was a negative predictor for achieving FC < 250 mg/kg (*p* = 0.045). *CYP24A1* 22776 CT and *VDR* Cdx2 GG increased the likelihood of presenting CD over UC (OR 3.40, *p* = 0.009 and OR 3.74, *p* = 0.047, respectively). Additionally, *CYP27B1* −1260 GT and +2838 CT increased the likelihood of non-ileal CD (OR 3.13, *p* = 0.054; OR 7.02, *p* = 0.01). **Conclusions**: This study reveals associations between VD-SNPs, clinical response to VDZ and UST, and IBD phenotype and localization, supporting the development of personalized IBD treatment and warranting further validation.

## 1. Introduction

Recent research has increasingly highlighted the role of vitamin D (VD) in the pathogenesis of various diseases, including inflammatory bowel disease (IBD), a group of chronic inflammatory conditions primarily affecting the bowel with systemic involvement [1]. VD is a critical regulator of immune responses and plays a key role in maintaining intestinal epithelial integrity and modulating the gut immune system [2]. In dextran sodium sulfate (DSS)-induced colitis in mice, selective deletion of the intestinal VD receptor (VDR) exacerbates colitis severity, leading to increased mucosal infiltration of Th1 and Th17 cells and a heightened inflammatory cytokine profile [3]. Observational studies suggest that low serum 25(OH)D3 levels are linked to a higher risk of IBD, reduced VDR expression (by approximately 50%) in the colonic epithelium of IBD patients compared to healthy controls, and a protective effect against *Clostridioides difficile* infection in those with higher 25(OH)D3 levels [4].

Pharmacogenomics, already widely utilized in oncology, HIV treatment, and autoimmune disorders, is gaining traction in IBD management, with a growing focus on VD due to its involvement in the transcriptional regulation of over 200 genes [5,6]. Recent studies have explored whether genetic variants in the VD pathway can predict susceptibility or different outcomes in immune-mediated or autoimmune diseases, including IBD [1]. The goal is to personalize treatment by identifying factors that influence disease progression and treatment response variability among patients. Predictors of clinical response are being sought through microbiological, immunological, genetic, and other analyses [7].

Evidence suggests an inverse correlation between pre-treatment VD levels and IBD activity, both at endoscopic as well at histological levels [8]. Generally, patients with moderate disease activity often have VD levels below 25 ng/mL. In addition to disease status, a correlation has been observed with the response to biologics, particularly adalimumab and vedolizumab (VDZ). A recent study on VDZ-treated patients found that VDR and CYP24A1 (the VD-inactivating enzyme) expression correlates with altered expression of the α4 and β7 subunits, affecting the inflammatory severity and the risk of therapy failure within one year [9]. These findings are consistent with VDZ’s mechanism of action as a selective α4β7 integrin blocker, which targets T-cell trafficking to the gut. Another study on adalimumab-treated patients highlighted the role of VD-related single nucleotide polymorphisms (SNPs) in predicting clinical remission at 3 and 12 months of treatment. Specifically, *CYP27B1* −1260 TT was associated with higher baseline fecal calprotectin (FC) (an inflammation marker) and lower hemoglobin levels. VDR BsmI AA genotype was identified as a negative predictor at 3 months, while the GC 1296 AA/AC genotype was a negative predictor at 12 months [10].

There is limited evidence on the relationship between VD pathway SNPs and response to ustekinumab (UST), an anti-IL 12/23 antibody. Understanding the function of SNPs and genotypes in genes involved in VD synthesis, transport, catabolism, and activity is crucial for the prediction of drug efficacy. This knowledge enables more precise customization of IBD treatments by tailoring them to the individual’s VD-related genetic profile.

In this context, the aim of the present study was to evaluate the role of VD-related genetics in predicting therapeutic response to VDZ and UST in patients with Crohn’s disease (CD) and ulcerative colitis (UC) after 12 months of therapy (T12).

## 2. Materials and Methods

This study was a two-center prospective study performed at the IBD unit of the Gastroenterology Department of the AOU Città della Salute e della Scienza of Turin, Italy, and at the University Hospital of Pisa, Italy. Patients were enrolled from July 2016 to December 2022. The study followed the principles of the Declaration of Helsinki and was approved by the local ethical committees: Comitato Etico Interaziendale A.O.U. Città della Salute e della Scienza di Torino—A.O. Ordine Mauriziano—A.S.L. Città di Torino (approval code 0056924); Comitato Etico Regionale Toscana Area Vasta Nord Ovest—CEAVNO (approval code 16790), approved on 8 June 2016.

### 2.1. Population

The initial sample consisted of 105 patients: 65 of them were treated with UST, whereas 40 were treated with VDZ. Two patients treated with UST did not attend follow-up visits at T3 and T12 and were thus excluded from the study. This brought the total number of patients included in the study to 103.

#### 2.1.1. Inclusion Criteria

We included individuals aged 18 years or older, diagnosed with IBD (either CD or UC) according to ECCO criteria [11,12], and with an indication to start treatment with VDZ or UST due to their inflammatory bowel disease. Patients were followed by the IBD outpatient clinic of the AOU Città della Salute e della Scienza of Turin and the AOU of Pisa. They provided informed consent, and they were evaluated at the time of biological drug initiation (T0), at 3 months after the start of administration (T3), and at 12 months (T12). At each of these outpatient visits, the medical history was collected, an abdominal physical examination was performed, and blood and stool laboratory tests were taken to correlate the clinically investigated disease activity with the reported values.

#### 2.1.2. Exclusion Criteria

As this is a real-life study, no exclusion criteria were applied concerning concomitant medications. However, patients initiating VDZ or UST primarily for other comorbidities (e.g., psoriatic arthritis) were excluded. Additionally, patients who transferred their follow-up care to other hospitals or discontinued follow-up visits were also excluded from the study.

### 2.2. Considered Parameters

The variables considered included patient characteristics, disease features, disease scores, as well as laboratory parameters. Both clinical and non-clinical data were analyzed to determine drug response outcomes at T3 and T12. In parallel, SNPs of specific genes in the VD pathway were analyzed for each patient to explore potential correlations between genetics and drug response. Specifically, patient characteristics included the following: age at start of treatment, gender, and smoking habits (including ex-smokers). Regarding disease characteristics, the following factors were considered: CD or UC; years of illness; surgical history; immune-mediated diseases associated with IBD; and disease localization expressed using the Montreal classification. For CD: ileal (L1), colic (L2), ileo-colic (L3), and upper gastroenteric tract (L4) localization. For UC: ulcerative proctitis (E1), ulcerative colitis distal to the splenic flexure, then left colitis (E2), and extensive ulcerative colitis (E3). At each time-point (T0, T3, and T12), clinical disease activity was assessed with Harvey–Bradshaw Index (HBI) for CD [13] and with Partial Mayo Score (PMS) for UC [14,15]. The HBI is based on five exclusively clinical parameters, each associated with a specific score; the sum of these scores defines the disease status: remission (score < 5), mild activity (5–7), moderate activity (8–16), and severe activity (score > 16). The PMS considers only the clinical parameters of the Full Mayo Score (stool frequency, rectal bleeding, and physician’s global assessment), with a total score of 9 points; a clinical response is defined as a decrease of at least 2 points in the PMS. According to this score, disease is considered in remission with a score of less than 2, mild activity between 2 and 4, moderate activity between 5 and 7, and severe activity with a score greater than 7. As laboratory parameters, C-reactive protein (CRP) and FC were assessed at each time-point by taking blood and stool samples. In the study, CRP was considered negative when <5 mg/L. FC has been utilized as a surrogate marker of mucosal healing [16,17].

### 2.3. Analysis

The DNA extraction protocol of the samples was a manual extraction performed following the extraction protocol provided by the DNA extraction kit (Qiagen, Valencia, CA, USA) proteinase K (inhibits nucleases that degrade DNA), AL buffer (lysis buffer), ethanol, AW1 and AW2 buffers (for washing), buffer AE (for elution), column and column holders, and sterile Eppendorf 1.5 mL (Hamburg, Germany). The procedure consisted of assigning a unique identification number to each patient and then adding 20 µL proteinase K and 200 µL blood to the sample column used for each. This was followed by the addition of 200 µL of Buffer AL to each column, vertexing for at least 15 s, incubation at 56 °C for 10 min, and centrifugation at 8000 rpm for one minute. Then it proceeded to the second step of the process by adding 200 µL ethanol per sample, vertexing again for 10 s, and centrifugation at 8000 rpm for one minute.

After that, the sample was transferred to a new column, 500 µL of Buffer AW1 was added and then centrifuged at 8000 rpm for one minute, and the column holder was replaced. After replacement, 500 µL of Buffer AW2 was added and centrifuged at 14,000 rpm for three minutes and finally the sample was transferred to an Eppendorf. Then 200 µL of Buffer AE was added and the sample was incubated at room temperature for one minute. Once the final washing steps were performed, the sample was centrifuged for one minute at 8000 rpm and placed at −80 °C.

Regarding SNP analysis of VD, allelic analyses were performed by polymerase chain reaction (PCR). The VD related genes analyzed were *CYP24A1* (coding for the enzyme responsible for the production of the inactive metabolite 24,25(OH)D3) 22776 CT, 8620 A>G, 3999 T>C; *VDR* (coding for the VD receptor) Apal C>A, Tag/T>C and Bsml G>A, Cdx2 A>G; *CYP27B1* (coding for the enzyme responsible for the production of 1,25VitD) −1260 G>T, +2838 C>T; *GC* (coding for VDBP or VD binding protein, its transporter) 1296 A>C. For PCR-real time analysis of the samples, the CFX96 Touch Real-Time PCR system from Bio-Rad Laboratories was used. The PCR protocol included the following: 10 min cycle at 95 °C; 30 s cycle at 50 °C; 15 s cycle at 95 °C; and 40 s cycle at 60 °C with plate reading. These cycles were performed successively on a volume of 25 µL per well, with a total duration of two hours and ten minutes. Fluorescent probes FAM (6-carboxyfluorescein) and VIC (4′,7′-dimethoxy-4-oxy-ROX) based on the principle of fluorescence resonance energy transfer (FRET) were used for signal detection. During amplification, DNA was replicated, and FAM or VIC molecules were incorporated into the new strands. When hit by a light beam, the molecules absorb energy and enter an excited state. If FAM and VIC are close together, energy can be transferred from FAM to VIC via FRET. VIC emits fluorescence at a longer wavelength, which is detected and recorded by the PCR instrument.

The Biorad CFX Manager 3.1 (Hercules, CA, USA) controls the instrument and processes the data, interfacing with the PC. The plates used were the Lightcycler^®^ 480 Multiwell Plate 96, manufactured by Roche (Monza, Italy).

The analysis of SNPs was conducted following an allelic discrimination protocol using water (H_2_O), master mix, TaqMan probes produced by Thermo Fisher Scientific specific company (Waltham, MA, USA) for each SNP tested and patient DNA; each protocol belongs to one of three macro-groups divided according to variable reagent volumes.

For preparation, specific volumes of H_2_O, master mix and probes were pipetted into an Eppendorf, then this mix was distributed into the wells of the plate at a constant volume according to the total volume of the protocol. The prepared plate was then transferred from the hood to the counter, where 2.5 µL of DNA extracted from each well was added, allocating one DNA sample per patient for each well. Finally, the plate was sealed and inserted into the PCR machine to complete the analysis.

The therapy-related information considered included the use of biological drugs (VDZ or UST), any administration of low-absorption glucocorticoids such as budesonide and beclomethasone dipropionate, and systemic glucocorticoids such as prednisone and methylprednisolone.

### 2.4. Outcomes

The outcomes in this study can be classified into primary and secondary.

The primary outcome was the clinical response at T12 to the administered drug (UST or VDZ). For CD, according to the International Organization for the Study of Inflammatory Bowel Disease (IOIBD), a clinical response is defined as a reduction of at least 3 points in the HBI from baseline (T0) or maintenance of remission (HBI < 5) between time points, or a transition from a higher to a lower disease activity (e.g., from moderate to mild), in patients with glucocorticoids withdrawal and without discontinuation of biological therapy. For UC, in accordance with IOIBD, clinical response is defined as the presence of a reduction of at least 2 points in PMS compared to the score before the start of therapy (T0), or maintenance of remission from one time to the next (PMS < 2), or change from a higher to a lower degree of activity (e.g., from moderate to mild activity) in patients with glucocorticoids withdrawal and without discontinuation of biological therapy.

The secondary outcomes were T3 clinical response to the drug (considering the same criteria as above to affirm any response) and achievement of FC < 250 mg/kg at T12, a surrogate marker of mucosal healing [16,17].

Patients who discontinued biological therapy within 12 months due to treatment failure or adverse events were classified as non-responders and included in the group that did not achieve the primary outcome. Furthermore, this group was excluded from the subanalyses of the secondary outcome, which assessed FC levels at T12.

### 2.5. Statistical Analyses

Categorical variables were indicated with a number (n) indicating the absolute value or a frequency expressed as a percentage. For continuous variables, the distribution was tested with the D’Agostino–Pearson test and variables with a normal distribution were reported as mean ± standard deviation (SD), while those with a non-normal distribution were reported as median and interquartile range (IQR). Comparisons of independent categorical variables were performed using the Chi-square test, followed by univariate logistic regression to assess the strength of associations with odds ratios (OR) and 95% confidence intervals (CI). Comparisons of independent continuous variables were conducted using the *t*-test for independent samples.

For all analyses, a *p*-value < 0.05 was considered statistically significant.

After compiling the data into a dedicated Excel database, statistical analysis was performed using MedCalc^®^ version 20.104.

## 3. Results

### 3.1. Descriptive Analysis of the Variables Considered

The baseline characteristics of the included patients are summarized in Table 1.

### 3.2. Laboratory Parameters Descriptive Analysis

The baseline C-reactive protein (CRP) values of included patients are reported in Table 1. By T3, the number of patients with negative CRP increased to 53 out of 100, with a corresponding decrease in those with CRP > 5 mg/L to 47 out of 100. At T12, protein evaluation was available for 95 patients, of whom 63.2% had a negative CRP and 36.8% had a positive result.

### 3.3. Descriptive Treatment Analysis

Another parameter assessed was the need for low-absorbed or systemic glucocorticoids during therapy, with the aim of discontinuing glucocorticoids use through biologic treatment. In the overall sample, 52.4% (*n* = 54) did not require glucocorticoids, whereas 47.6% (n = 49) did.

### 3.4. Outcomes Descriptive Analysis

As previously mentioned, the primary outcome of the study was achieving a clinical response at T12, in patients with glucocorticoid withdrawal and without discontinuation of biological therapy. This outcome was reached in 78.6% of cases (n = 81 out of 103). The secondary outcome of clinical response at T3 was achieved in 73.8% of cases (n = 76). Of the 91 out of 103 patients for whom FC data were available at T12, 71.4% (n = 65) achieved the secondary outcome of an FC level < 250 mg/kg. Additionally, a significant finding related to achieving FC levels below 250 mg/kg was the reduction in calprotectin between T0 and T3, observed in 82.1% of cases (n = 78 out of 95), with no reduction seen in 17.9% (n = 17).

### 3.5. Associations and Logistic Regression Between the Variables and Outcomes Considered

Associations between various parameters and clinical response at T12 were analyzed to identify any significant correlations (Table 2). While many of the variables analyzed did not reach statistical significance, the relationship between clinical response at T3 and the clinical response at T12 showed a significant *p*-value of 0.0002.

**Figure 1:**
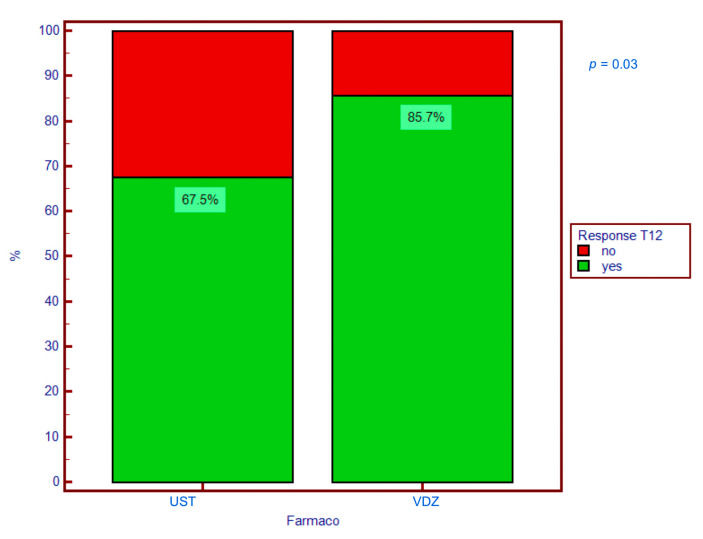
Clinical response at T12 for UST and VDZ. T12 = clinical response at 12 months; UST = ustekinumab; VDZ = vedolizumab.

Ustekinumab showed greater efficacy in achieving the primary outcome, with 85.7% of patients responding at T12 versus 67.5% with vedolizumab (OR 2.89, CI 1.10–7.6, *p* = 0.03) (Figure 1).

We also analyzed the relationship between FC reduction from T0 to T3 and achieving FC levels < 250 mg/kg at T12, which demonstrated a statistically significant association (OR 5.22, 95% CI 1.76–15.30, *p*-value < 0.0001). In contrast, the correlation between FC reduction from T0 to T3 and clinical response at T12 was not statistically significant (OR 2.15, 95% CI 0.78–5.97, *p* = 0.14), suggesting that this parameter was predictive only of achieving FC levels < 250 mg/kg at T12. Additionally, we examined the correlation between achieving FC levels < 250 mg/kg at T12 and disease duration. Subjects who did not achieve the outcome had a mean disease duration of 15.7 years, compared to 17.7 years in those who achieved the outcome, with no statistically significant difference (*p*-value = 0.37).

### 3.6. Descriptive Analysis of SNPs

Table 3 and Table 4 display the distribution of wild type (WT), heterozygous, and homozygous mutated genotypes, along with their corresponding percentages across the entire sample.

### 3.7. Associations Between Study Variables and Gene Polymorphisms

A key component of the statistical analysis in this study involved cross-referencing genetic data with patients’ clinical and laboratory responses. All genes, including their SNPs, were assessed and correlated with the T12 response. While most analyses did not reveal statistically significant correlations capable of predicting clinical response or non-response based on genotype, one notable finding emerged for the GC gene at position 1296, where an A>C nucleotide substitution occurs. This gene, which encodes the VD transporter protein, demonstrated that among non-responders at T12, 63.6% possessed the heterozygous AC genotype. In contrast, those who achieved the desired clinical outcome exhibited a more balanced distribution of genotypes.

Given the significant association, this gene and its corresponding SNPs were further analyzed in relation to the response at T12 for both biological drugs. In the subset of patients treated with VDZ, the results indicated that heterozygous individuals exhibited a less favorable response to therapy, with only 46.7% achieving the primary outcome. The analysis yielded an OR of 4.57 (95% CI: 1.12–18.73, *p* = 0.04), confirming a reduced therapeutic response in this group.

*CYP24A1* 8620 A>G was investigated for its correlation with achieving FC levels < 250 mg/kg at T12. Although the Chi-square test showed no statistically significant association between the overall genotypes and the outcome (*p* = 0.08), logistic regression analysis revealed a significant association for the heterozygous AG genotype, with a *p*-value of 0.045. This suggests that the AG genotype reduces the likelihood of achieving calprotectin levels < 250 mg/kg at T12, thus serving as a negative predictor. The highest proportion of individuals failing to reach FC levels < 250 mg/kg were heterozygous. Specifically, among the 26 patients with FC ≥ 250 mg/kg at T12, 13 were heterozygous. Furthermore, it is noteworthy that the majority of homozygous mutated subjects, 82.9% (n = 29 out of 35), successfully achieved the outcome.

**Figure 2:**
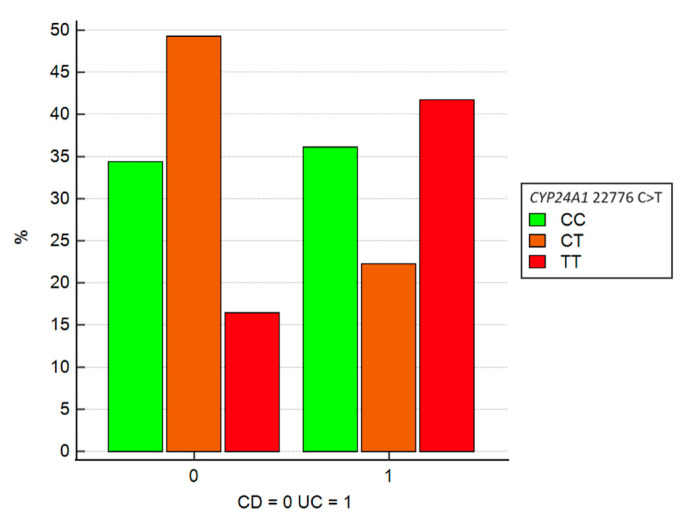
Graphical representation of logistic regression between the CYP24A1 22776 SNP and disease distribution (CD or UC). SNP = single nucleotide polymorphism; CD = Crohn’s disease; UC = ulcerative colitis.

The *CYP24A1* 22776 C>T gene demonstrated a significant association with the categorical disease variable ‘CD or CU’ according to Chi-square testing (*p*-value = 0.006). This suggests that the distribution of IBD types differs significantly among the three genotypes. Univariate logistic regression analysis indicated that the heterozygous CT genotype (represented as 1 in Figure 2) was associated with an approximately 3-fold increased likelihood of CD compared to other genotypes (OR = 3.40, 95% CI: 1.35–8.52, *p*-value = 0.009).

**Figure 3:**
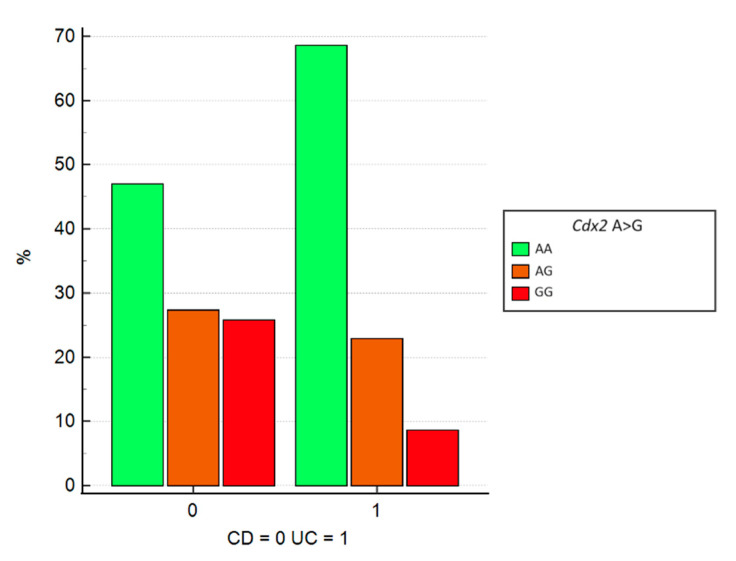
Graphical representation of logistic regression between SNPs of the VDR Cdx2 gene and disease distribution (CD or CU). SNP = single nucleotide polymorphism; VDR = vitamin D receptor; CD = Crohn’s disease; UC = ulcerative colitis.

The association between the VDR Cdx2 gene mutation, A>G nucleotide substitution, and the onset of CD or UC revealed a significant difference in the distribution of IBD phenotypes across genotypes (*p* = 0.019). Additionally, logistic regression analysis identified the homozygous GG mutation as being associated with a 3.7-fold increased risk of developing CD compared to UC (OR = 3.74, 95% CI = 1.02–13.77, *p* = 0.047). Figure 3 illustrates that 25.8% of patients with CD are homozygous for the GG mutation, compared to only 8.6% of those with UC. Moreover, among all patients with the GG genotype, 85% have CD, while only 15% have UC.

Lastly, we explored the potential correlation between genotypes and ileal involvement in CD (L1). Chi-square tests indicated that CD localization varies according to CYP27B1 gene genotypes. Specifically, analysis of SNPs at positions −1260 (G>T) and +2838 (C>T) in the *CYP27B1* gene revealed statistically significant associations, with *p*-values of 0.047 and 0.025, respectively.

Univariate logistic regression was subsequently performed for both mutation sites (Table 5). Regarding *CYP27B1* −1260 GT, heterozygous individuals with this mutation exhibit a 3.1-fold increased likelihood of having non-ileal CD (non L1-CD), though the *p*-value approached the threshold for significance (OR = 3.13, 95% CI: 0.98–10.01, *p* = 0.054). Heterozygous carriers of *CYP27B1* +2838 CT have a 7-fold increased probability of non L1-CD (OR = 7.02, 95% CI: 1.44–34.25, *p* = 0.01).

**Figure 4:**
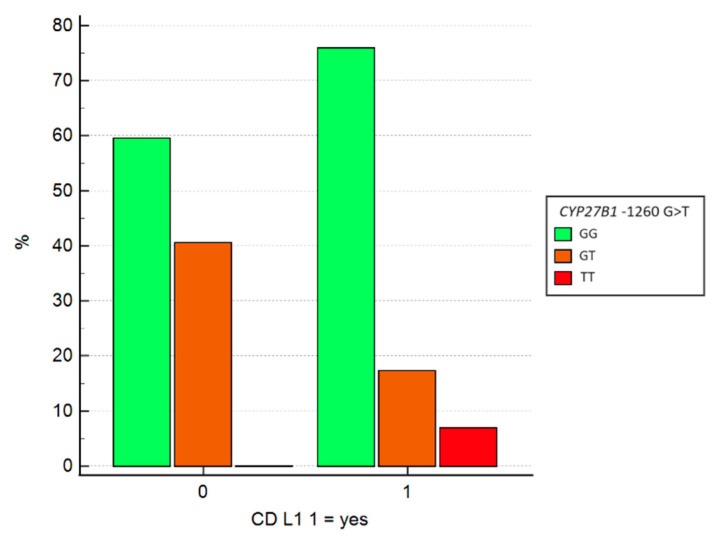
Logistic regression between SNPs of the CYP2781 gene in −1260 and CD localization. SNP = single nucleotide polymorphism; CD = Crohn’s disease.

Figure 4 shows that 40.5% of patients with non L1-CD are heterozygous for the GT mutation, while only 17.2% of patients with L1-CD have this mutation.

**Figure 5:**
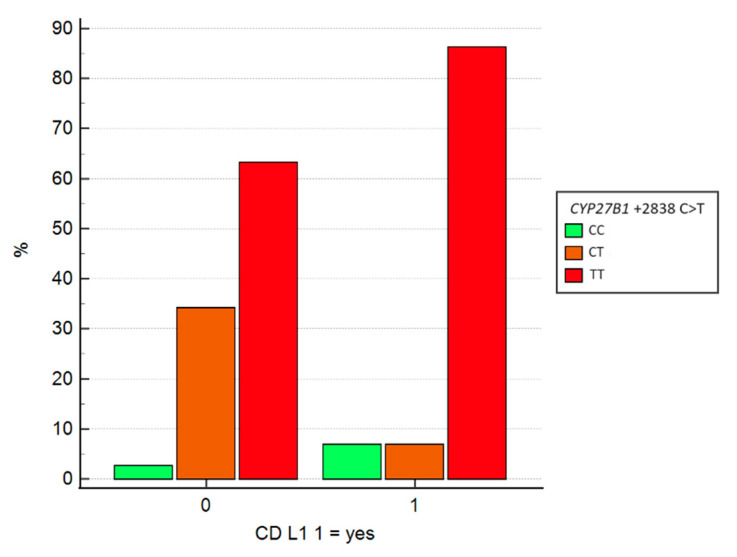
Logistic regression between SNPs of the CYP2781 gene in +2838 and CD localization. SNP = single nucleotide polymorphism; CD = Crohn’s disease.

Figure 5 illustrates that among patients with the CT mutation, the majority have non-ileal CD, with 34.2% presenting with non-L1 CD and only 6.9% with L1 CD.

## 4. Discussion

VDZ and UST are two biologic agents widely used in IBD treatment. Predicting their efficacy in reducing disease activity and maintaining remission in CD and UC is an ambitious but crucial goal for optimizing patient-specific therapeutic strategies. This study investigates whether the therapeutic response at 12 months of treatment with these biologics could be influenced by or correlated with allelic variants in VD pathway genes. This investigation is grounded in existing literature that suggests the potential predictive role of VD in certain autoimmune and immune-mediated diseases, given its ability to modulate the immune system and regulate pro-inflammatory cytokines driving inflammation [1]. Moreover, VD is known to downregulate integrin α4β7, which has been implicated in the response to VDZ [9]. Finally, VD is able to affect the expression of gene encoding enzymes and the transporter involved in drug metabolism end transport, impacting on drug concentrations as shown for different drugs [18].

The study also explored associations between genetic SNPs and disease type (CD or UC), disease extent, and the relationship between various gene SNPs and the secondary outcome of FC levels < 250 mg/kg.

### 4.1. Clinical and Laboratory Variables

The literature presents a range of studies that either support or challenge the predictive value of patient-specific and disease-specific clinical and laboratory variables for the response to VDZ and UST in IBD. However, fewer studies are currently available for UST. In the present study, smoking habits did not significantly influence clinical and laboratory outcomes. This finding contrasted with the limited data from the US-VICTORY cohort, which found that a smoking history was associated with a lower rate of clinical remission at 12 months of VDZ treatment. Further research is required, as its role in biologic response remains unclear [19]. The impact of baseline disease severity on treatment outcomes in patients treated with VDZ and UST has been extensively investigated [20,21,22]. In contrast with current data from literature, our study found no statistically significant correlation between baseline moderate–severe and clinical response or FC levels < 250 mg/kg at T12. Our study identified a statistically significant association between a reduction in FC levels from T0 to T3 and FC levels < 250 mg/kg at T12 and (OR = 5.23, 95% CI: 1.79–15.29, *p*-value = 0.003). A substantial body of evidence supports FC levels and their reduction during therapy as predictors of biologic response, though most studies focus on anti-TNF agents [23]. The study did not find a statistically significant influence of disease type (CD or UC) on treatment response at T12, which aligns with other studies investigating VDZ in IBD [24]. However, in our study, response rates at T12 varied depending on the biologic used, with UST demonstrating statistically significant superiority in terms of efficacy.

### 4.2. Polymorphisms in VD Pathway Genes

In our study, we investigated SNPs in several genes involved in the VD pathway and associated them with clinical response to VDZ and UST at T3, T12, FC levels < 250 mg/kg at T12, as well as with the type and localization of IBD.

The most notable findings concern the GC gene, encoding the VD transporter, specifically the A>C polymorphism at position 1296. We found that patients with the AC heterozygous genotype had a reduced response to biological therapy with VDZ and UST at T12 compared to patients with the WT AA or homozygous CC genotype, suggesting this could be a negative predictor of response at T12. Among patients receiving VDZ, this genotype significantly predicted a reduced response at T12 (OR 4.57, 95% CI: 1.12–18.73, *p* = 0.04). This was in contrast with a previous study by the same teams in Turin and Pisa, where the same genotype was a positive predictor of response to adalimumab [10]. Future studies should further explore the role of the AC genotype, as it might play a key role in the choice of biologic therapy. Notably, all homozygous CC patients in our study achieved response at T12, but due to the small sample size (10 patients), further research is needed to draw meaningful conclusions.

We also investigated the *CYP24A1* gene, which inactivates VD3 (cholecalciferol) to calcitroic acid. The A>G polymorphism at position 8620 was analyzed in this context. While literature suggests a link between *CYP24A1* rs6013905AX and rs2762939GX SNPs and colorectal cancer (*p* ≤ 0.05) [6], little is known about their relevance to IBD. In our study, the AG genotype was a negative predictor of achieving FC levels < 250 mg/kg after 12 months of VDZ and UST therapy (OR 0.38, 95% CI: 0.15–0.98, *p* = 0.046). Unexpectedly, 82.9% (29/35) of homozygous GG patients reached FC < 250 mg/kg at T12.

Additionally, we assessed another polymorphism in *CYP24A1*, 22776 C>T, but no significant associations were observed for our predefined outcomes, consistent with a previous study on adalimumab [10]. However, we found an association between the CT genotype and CD vs. UC, with a *p*-value of 0.009 (OR 3.74, 95% CI: 1.35–8.52), indicating that carriers of the CT genotype have approximately three times the likelihood of developing CD compared to UC.

We also analyzed the A>G polymorphism in the Cdx2 gene, which encodes the VDR. This gene activates transcription factors that stimulate genes involved in immune homeostasis, metabolism, absorption of ions such as calcium and phosphorus, and the apoptotic mechanism. In our study, the homozygous GG genotype was associated with an approximately threefold increased likelihood of developing CD compared to UC (OR 3.74, 95% CI: 1.02–13.77, *p* = 0.047). Existing literature already indicates a correlation between Cdx2 SNPs and the onset of inflammatory bowel disease [25]. However, there is no significant evidence linking Cdx2 specifically to one type of IBD over another.

Our study investigated two SNPs in the gene encoding the VD-activating enzyme, CYP27B1: −1260 G>T and +2838 C>T, aiming to correlate them with the extent of CD. Previous studies demonstrated a significant association between VD-related genetic mutations and CD localization. Notably, a study conducted by the Turin group examined the same SNPs and their correlation with perianal Crohn’s disease (pCD). The study found that heterozygosity for VDR ApaI and BsmI SNPs significantly increased the risk of pCD [26]. Building on these findings, we assessed the likelihood of non-ileal CD (non-L1) presentation in relation to the studied SNPs. The *CYP27B1* gene revealed a threefold increased probability of non-ileal CD in patients with the GT genotype at −1260 and up to a sevenfold increased probability for those with the heterozygous CT genotype at +2838.

The main limitation of the present study is the sample size. Despite analyzing 103 patients, the number of homozygous mutants for most SNPs was insufficient to draw definitive conclusions. Additionally, some of our statistical analysis failed to achieve significance, likely due to the limited sample size, highlighting the need for larger populations in future studies to improve statistical power. Finally, we did not account for endoscopic findings at different time points, which could provide a more reliable indicator for mucosal healing at T12. However, several studies have demonstrated the correlation between FC levels < 250 mg/kg and mucosal healing in IBD patients [16,17].

## 5. Conclusions

In recent years, genetic analysis has become increasingly pivotal in predicting responses to biologic therapies in IBD. Pharmacogenomics, already established in other fields, is gaining attention in IBD management, with a growing focus on VD due to its role as an immunoregulatory factor and its involvement in the transcriptional regulation of over 200 genes. Our findings identified some potential associations between specific SNPs and clinical and biochemical outcomes. This result underscores the potential for genotype-based selection of biologic therapy, if our results would be validated by future research with larger patient sample size.

## Figures and Tables

**Table 1:** Descriptive analysis of baseline patient characteristics.

Baseline Characteristics	
Sex—% (n/tot)	
Females	35.9 (37/103)
Males	64.1 (66/103)
Age—median (IQR)	53 (42–66)
Smoking Habits—%(n/tot)	
Smokers	16.5% (17/103)
Non-smokers	71.8% (74/103)
Ex-smokers	11.7% (12/103)
CD Localization (L)—% (n/tot)	
L1	43.3% (29/67)
L2	14% (10/67)
L3	41.8% (28/67)
Total (L variables)	65% (67/103)
UC Localization (E)—% (n/tot)	
E1	13.9% (5/36)
E2	11.1% (4/36)
E3	75% (27/36)
Total (E variables)	35% (36/103)
Years of Disease—mean ± SD	16.9 ± 9.8
Surgery—% (n/tot)	
CD Surgery	52.2% (35/67)
CU Surgery	5.5% (2/36)
Disease Activity at T0	
CD (HBI)—median (IQR)	8.0 (6.0–11.0)
CU (PMS)—mean ± SD	4.9 ± 1.7
CRP Status at T0—% (n/tot)	
CRP < 5 mg/L	39% (39/100)
CRP > 5 mg/L	61% (61/100)
Faecal Calprotectin (T0)—median (IQR)	421 (239.8–856.5)
Treatment—% (n/tot)	
UST	61.2% (63/103)
VDZ	38.8% (40/103)

CD = Crohn disease; UC = ulcerative colitis; HBI = Harvey–Bradshaw Index; PMS = Partial Mayo Score; CRP = C-reactive protein (CRP); UST = ustekinumab; VDZ = vedolizumab.

**Table 2:** Univariate logistic regression analysis between considered variables and clinical response at T12. OR: odds ratio Cl: 95% confidence interval. NS = non-significant; * = *p*-value < 0.05.

Variables	OR	95% CI	*p*-Value
Sex	1.26	0.46–3.44	0.65 (NS)
Smokers	0.86	0.25–2.96	0.81 (NS)
Years of disease	0.99	0.95–1.04	0.74 (NS)
CD	1.76	0.67–4.60	0.25 (NS)
CU	2.22	0.47–10.57	0.32 (NS)
L1-CD	1.67	0.45–6.19	0.45 (NS)
UST	2.89	1.10–7.60	0.03 *
Steroid therapy at T0	0.55	0.21–1.44	0.23 (NS)
Degree of disease activity > 1 at T0	1.21	0.47–3.13	0.70 (NS)
FC reduction from T0 to T3	2.15	0.78–5.97	0.14 (NS)
Clinical response at T3	6.91	2.48–19.30	0.0002 *

**Table 3:** Polymorphisms of analyzed genes.

Genes	Wild Type	Heterozygous	Homozygous Mutation
*CYP24A1 22776 C>T*	CC	CT	TT
*CYP24A1 8620 A>G*	AA	AG	GG
*CYP24A1 3999 T>C*	TT	TC	CC
*VDR ApaI C>A*	CC	CA	AA
*VDR TaqI T>C*	TT	TC	CC
*VDR BsmI G>A*	GG	GA	AA
*VDR Cdx2 A>G*	AA	AG	GG
*GC A>C*	AA	AC	CC
*CYP27B1 −1260 G>T*	GG	GT	TT
*CYP27B1 +2838 C>T*	CC	CT	TT

**Table 4:** Descriptive analysis of the allelic distribution of genetic polymorphisms in the sample expressed in %.

Genes	Wild Type	%	Heterozygous	%	Homozygous Mutation	%
*CYP24A1 22776*	CC	35.0%	CT	39.8%	TT	25.2%
*CYP24A1 8620*	AA	26.2%	AG	36.9%	GG	35.9%
*CYP24A1 3999*	TT	24.5%	TC	50.0%	CC	25.5%
*VDR ApaI*	CC	28.2%	CA	26.2%	AA	45.6%
*VDR TaqI*	TT	43.6%	TC	30.7%	CC	25.7%
*VDR BsmI*	GG	37.9%	GA	36.9%	AA	25.2%
*VDR Cdx2*	AA	54.5%	AG	25.7%	GG	19.8%
*GC 1296*	AA	35.3%	AC	44.1%	CC	20.6%
*CYP27B1 −1260*	GG	68.3%	GT	28.7%	TT	3.0%
*CYP27B1 +2838*	CC	5.9%	CT	23.5%	TT	70.6%

**Table 5:** Univariate logistic regression analysis of the association between genetic polymorphisms and clinical and laboratory outcomes, and the correlation between genetic polymorphisms and disease type with associated localization. T12 = clinical response at 12 months; VDZ= vedolizumab; FC = fecal calprotectin; CD = Crohn’s disease; NS = non-significant; * = *p*-value < 0.05.

	Response at T12			No Response After 12 Months of VDZ			Failure to Achieve FC < 250 mg/kg at T12			CD			CD Extension: Non-Ileal CD		
	OR	CI	*p*-Value	OR	CI	*p*-Value	OR	CI	*p*-Value	OR	CI	*p*-Value	OR	CI	*p*-Value
*CYP24A1* 22776 CT	0.74	0.29–1.93	0.54 (NS)							3.4	1.35–8.52	0.009			
*CYP24A1* 22776 TT	0.66	0.23–1.85	0.43 (NS)												
*GC* 1296 AC				4.57	1.12–18.73	0.04 *									
*CYP24A1* 8620 AG							0.38	0.15–0.98	0.046 *						
*VDR Cdx2* GG										3.74	1.02–13.77	0.047 *			
*CYP27B1* −1260 GT													3.13	0.98–10.01	0.054 (NS)
*CYP27B1* +2838 CT													7.02	1.44–34.25	0.01 *

## Data Availability

The data presented in this study are available on request from the corresponding author.
